# Transcriptional profiling of *PRKG2*-null growth plate identifies putative down-stream targets of *PRKG2*

**DOI:** 10.1186/s13104-015-1136-6

**Published:** 2015-04-30

**Authors:** James E Koltes, Dinesh Kumar, Ranjit S Kataria, Vickie Cooper, James M Reecy

**Affiliations:** Department of Animal Science, Iowa State University, 2255 Kildee Hall, Ames, IA 50011 USA; National Bureau of Animal Genetic Resources, Karnal, 132001 Haryana India; Veterinary Diagnostics and Production Animal Medicine, Iowa State University College of Veterinary Medicine, Ames, IA 50011-3150 USA; Current address: Centre for Agricultural Bioinformatics, Indian Agricultural Statistics Research Institute, Library Avenue, PUSA, New Delhi, 110012 India

**Keywords:** Cattle, cGMP-dependent, Type II, Protein kinase (PRKG2), Dwarfism

## Abstract

**Background:**

Kinase activity of cGMP-dependent, type II, protein kinase (PRKG2) is required for the proliferative to hypertrophic transition of growth plate chondrocytes during endochondral ossification. Loss of PRKG2 function in rodent and bovine models results in dwarfism. The objective of this study was to identify pathways regulated or impacted by PRKG2 loss of function that may be responsible for disproportionate dwarfism at the molecular level.

**Methods:**

Microarray technology was used to compare growth plate cartilage gene expression in dwarf versus unaffected Angus cattle to identify putative downstream targets of PRGK2.

**Results:**

Pathway enrichment of 1284 transcripts (nominal p < 0.05) was used to identify candidate pathways consistent with the molecular phenotype of disproportionate dwarfism. Analysis with the DAVID pathway suite identified differentially expressed genes that clustered in the MHC, cytochrome B, WNT, and Muc1 pathways. A second analysis with pathway studio software identified differentially expressed genes in a host of pathways (e.g. CREB1, P21, CTNNB1, EGFR, EP300, JUN, P53, RHOA, and SRC). As a proof of concept, we validated the differential expression of five genes regulated by P53, including *CEBPA*, *BRCA1*, *BUB1*, *CD58*, and *VDR* by real-time PCR (p < 0.05).

**Conclusions:**

Known and novel targets of PRKG2 were identified as enriched pathways in this study. This study indicates that loss of PRKG2 function results in differential expression of P53 regulated genes as well as additional pathways consistent with increased proliferation and apoptosis in the growth plate due to achondroplastic dwarfism.

**Electronic supplementary material:**

The online version of this article (doi:10.1186/s13104-015-1136-6) contains supplementary material, which is available to authorized users.

## Background

PRKG2 is a key regulatory kinase in the proper temporal and spatial development of growth plate cartilage. Loss of PRKG2 function results in a disorganized mixture of hypertrophic and proliferative chondrocytes instead of the highly organized columnar array observed in properly formed growth plates [1]. Mouse knockouts and natural mutations in *PRKG2* in rats and cattle exhibit achondroplastic dwarfism [1-3]. A deletion of *PRKG2* on human chromosome 4q21 was recently associated with growth restriction and mental retardation [4]. Thus, the functional role of PRKG2 in growth plate development is highly conserved across species. Based on the impact of PRKG2 on growth plate elongation across species, it is likely that many of the pathways regulated downstream of PRKG2 are also highly conserved.

Several targets of PRKG2 have been identified previously. PRKG2 signaling regulates growth plate chondrocyte hypertrophy and hyperplasia via SRY, sex determining region Y, −box 9 (SOX9), glycogen synthase 3 beta (GSK3b), and additional unknown factors [2,5,6]. PRKG2 phosphorylation is required for nuclear translocation of SOX9. Phosphorylated SOX9 modulates collagen expression from a proliferative (collagen 2, *COL2*) to a hypertrophic (collagen 10, *COL10*) program [2]. It is currently unclear whether PRKG2 directly or indirectly regulates SOX9 signaling [6]. Phosphorylation of GSK3b at Serine 9 by a protein complex comprised of PRKG2 and Axin1 causes increased beta-catenin (CTNNB1) activity and increased hypertrophy of growth plate chondrocytes [5]. Haploinsufficiency of *GSK3b* was capable of 30-40% rescue of skeletal growth in *PRKG2*^*−/−*^ mice [5].

Several regulators of PRKG2 have also been identified. Notable regulators of PRKG2 include C-type naturetic peptide (CNP), cyclic guanosine monophosphate (cGMP) and nitric oxide (NO). CNP regulates multiple pathways required for proper endochondral ossification. Various mouse crosses have demonstrated that transgenic modulation of CNP can rescue mice from achondroplasia caused by disruption of fibroblast growth factor receptor 3 (FGFR3), mitogen-activated protein kinase (MAPK), or PRKG2 signaling [7-9]. However, no direct studies have been performed to determine if FGFR3 and MAPK are regulated by PRKG2. Additionally, cGMP and NO act as upstream triggers for PRKG2 signaling. These small molecules have been tied to calcium signaling, apoptosis, and proliferation processes [10,11] as well as regulation of MAPK, nuclear factor of kappa light polypeptide gene enhancer in B-cells (NF-κB), mitogen-activated protein kinase 8 (JNK/ MAPK8), vascular endothelial growth factor (VEGF), and cAMP responsive element binding protein 1 (CREB) signaling [12].

To date, no studies have dissected the role of *PRKG2* regulation of global gene expression in the growth plate. Our objective was to characterize transcriptional changes in pathways downstream of PRKG2 in hopes of discovering additional regulators responsible for the switch from proliferative to hypertrophic growth plate development. A secondary objective is to understand the changes in transcriptional networks in the growth plate due to achondroplastic dwarfism. Transcriptional profiling of Angus *PRKG2*^*R678X/R678X*^ (dwarf) versus *PRKG2*^*R678X/+*^ (unaffected) cattle was used as a means to identify downstream targets of PRKG2.

## Results

### Analysis of differential expression

No differentially expressed (DE) genes were detected across genotypes after correcting for multiple testing. Since the statistical power to detect differences in gene expression was limited, pathway analyses were used as a filter to prioritize DE genes. The rationale was that transcripts within a DE signaling pathway or biological process were likely to be over-represented even when all genes were not DE. A nominal p < 0.05 significance level was used for each photomechanical transfer unit (PMT) level to declare “significance” as a first filter for the data. We focused on pathway analysis of the PMT 70 DE gene list, because it appeared the most valid based on the p-value histogram. The PMT 80 and PMT 90 gene lists were used in combined analyses only to corroborate results from the PMT 70 DE gene list.

### Summary of nominally DE genes

Using a significance level of p < 0.05, 1284 genes were detected as DE at scanning PMT 70, 860 genes at PMT 80, and 628 genes at PMT 90. Overlap between all three PMT levels included 180 genes. A summary of the significant probes is provided in Additional file 1.

### DAVID enrichment results are consistent with known PRKG2 biology

The gene ontology (GO) classification of genes represented at PMT 70 showed the greatest over-representation of genes involved in apoptosis, cell cycle regulation, cell death, protein modification, and phosphorylation (p < 0.01, Family-wise false discovery rate (FWFDR) < 0.10) using the database for annotation, visualization and integrated discovery (DAVID) tool (Table 1). Terms over-represented from the Panther molecular function database included: non-receptor serine threonine protein kinase, nucleic acid binding, transferase, protein kinase, non-motor actin and guanyl-nucleotide exchange factor (p < 0.01, FWFDR < 0.10) (Table 2). Functionally enriched gene clusters with statistically significant enrichment score (ES) included: proliferation (ES = 2.9), actin cytoskeletal development (ES = 2.25), and differentiation and apoptosis (ES = 2.17). The Angiotensin II mediated activation of JNK Pathway via protein tyrosine kinase 2 beta (PYK2) dependent signaling pathway was over-represented in the Biocarta pathway database (p < 0.05). Over-represented KEGG pathways included: MAPK signaling, Adherens junction, and Glycan structures - biosynthesis 2 (p < 0.05).

**Table 1:** **Summary of Gene Ontology (GO) biological processes over-represented in the PMT70 data (p < 0.01, FWFDR < 0.10)**

**GO ID and Term**	**# genes**	**% total**	**P-value**	**Benjamini** ^*^
**GO:0048468 ~ cell development**	82	10.26%	0.0001	0.02
**GO:0048856 ~ anatomical structure development**	127	15.89%	0.0002	0.02
**GO:0043170 ~ macromolecule metabolic process**	357	44.68%	0.0004	0.02
**GO:0016043 ~ cellular component organization and biogenesis**	153	19.15%	0.0004	0.02
**GO:0008283 ~ cell proliferation**	56	7.01%	0.0004	0.01
**GO:0044238 ~ primary metabolic process**	400	50.06%	0.0010	0.03
**GO:0044237 ~ cellular metabolic process**	399	49.94%	0.0010	0.02
**GO:0019953 ~ sexual reproduction**	28	3.50%	0.0011	0.02
**GO:0007275 ~ multicellular organismal development**	132	16.52%	0.0011	0.02
**GO:0048869 ~ cellular developmental process**	107	13.39%	0.0011	0.02
**GO:0006928 ~ cell motility**	32	4.01%	0.0030	0.04
**GO:0051674 ~ localization of cell**	32	4.01%	0.0030	0.04
**GO:0009566 ~ fertilization**	9	1.13%	0.0042	0.05
**GO:0016265 ~ death**	53	6.63%	0.0053	0.06
**GO:0009605 ~ response to external stimulus**	43	5.38%	0.0056	0.06
**GO:0007049 ~ cell cycle**	56	7.01%	0.0056	0.06
**GO:0006950 ~ response to stress**	65	8.14%	0.0067	0.06
**GO:0022414 ~ reproductive process**	24	3.00%	0.0073	0.06

^*^Benjamini is a measure of family-wise false discovery rate as described by Huang da et al. [13].

**Table 2:** **Summary of Panther molecular function categories over-represented in the PMT70 data (p < 0.01, FWFDR < 0.10)**

**Panther molecular function process**	**# genes**	**% total**	**P-value**	**Benjamini** ^*^
**MF00213: Non-receptor serine/threonine protein kinase**	188	23.5	6.00E-10	1.40E-07
**MF00042: Nucleic acid binding**	299	37.4	7.40E-09	8.80E-07
**MF00131: Transferase**	113	14.1	1.50E-08	1.20E-06
**MF00108: Protein kinase**	75	9.4	1.30E-04	6.00E-03
**MF00262: Non-motor actin binding protein**	137	17.1	2.80E-04	1.10E-02
**MF00101: Guanyl-nucleotide exchange factor**	98	12.3	7.50E-03	9.00E-02

^*^Benjamini is a measure of family-wise false discovery rate as described by Huang da et al. [13].

Enriched biological processes from the combined analysis, including genes from the PMT70, PMT 80 and PMT 90 gene lists, were very similar to those found from the PMT 70 gene list (Table 3). Notable biological processes over-represented in this analysis include: apoptosis, cell cycle regulation, and cell death (p < 0.005, FWFDR < 0.10). Notably, wingless-type MMTV integration site family (WNT) signaling was over-represented in the combined analysis (p < 0.05) (Table 4). Functional gene clustering results were also similar to those observed in the PMT 70 data, including clusters enriched for proliferation (ES = 3.69) and differentiation and apoptosis (ES = 3.69) (Additional file 2). Note, an ES ≥ 1.3 is equivalent to p < 0.05. [13].

**Table 3:** **Summary of biological processes enriched when all three PMT are combined (p < 0.01, FWFDR < 0.10)**

**GO Term**	**# genes**	**% total**	**P-value**	**Benjamini** ^*^
**GO:0048522 ~ positive regulation of cellular process**	136	8.50%	1.85E-07	7.82E-05
**GO:0048518 ~ positive regulation of biological process**	149	9.31%	1.01E-07	8.51E-05
**GO:0048519 ~ negative regulation of biological process**	154	9.62%	4.53E-07	1.28E-04
**GO:0048523 ~ negative regulation of cellular process**	148	9.25%	6.73E-07	1.42E-04
**GO:0006996 ~ organelle organization and biogenesis**	152	9.50%	2.39E-06	4.03E-04
**GO:0048731 ~ system development**	207	12.94%	6.89E-06	9.69E-04
**GO:0030154 ~ cell differentiation**	210	13.12%	3.55E-05	4.27E-03
**GO:0008219 ~ cell death**	108	6.75%	4.13E-05	4.35E-03
**GO:0009892 ~ negative regulation of metabolic process**	64	4.00%	6.11E-05	5.71E-03
**GO:0051726 ~ regulation of cell cycle**	74	4.62%	7.60E-05	6.39E-03
**GO:0044260 ~ cellular macromolecule metabolic process**	369	23.06%	9.27E-05	7.09E-03
**GO:0009893 ~ positive regulation of metabolic process**	64	4.00%	1.12E-04	7.85E-03
**GO:0019538 ~ protein metabolic process**	381	23.81%	1.83E-04	0.012
**GO:0022402 ~ cell cycle process**	94	5.88%	4.13E-04	0.025
**GO:0007626 ~ locomotory behavior**	33	2.06%	5.76E-04	0.030
**GO:0042127 ~ regulation of cell proliferation**	67	4.19%	5.46E-04	0.030
**GO:0044248 ~ cellular catabolic process**	77	4.81%	6.41E-04	0.031
**GO:0006397 ~ mRNA processing**	40	2.50%	7.12E-04	0.033
**GO:0048513 ~ organ development**	146	9.12%	8.33E-04	0.036
**GO:0050790 ~ regulation of catalytic activity**	64	4.00%	1.33E-03	0.050
**GO:0006928 ~ cell motility**	57	3.56%	1.22E-03	0.050
**GO:0043067 ~ regulation of programmed cell death**	70	4.38%	1.30E-03	0.051
**GO:0009653 ~ anatomical structure morphogenesis**	129	8.06%	1.77E-03	0.063
**GO:0008380 ~ RNA splicing**	34	2.12%	2.95E-03	0.095
**GO:0003012 ~ muscle system process**	27	1.69%	3.08E-03	0.095
**GO:0051641 ~ cellular localization**	105	6.56%	2.85E-03	0.096
**GO:0051649 ~ establishment of cellular localization**	102	6.38%	3.37E-03	0.097
**GO:0006730 ~ one-carbon compound metabolic process**	18	1.12%	3.28E-03	0.098

^*^Benjamini is a measure of family-wise false discovery rate as described by Huang da et al. [13].

**Table 4:** **Summary of all pathways with over-represented gene lists from KEGG (p < 0.05)**

**KEGG pathway**	**# genes**	**% total**	**P-value**	**Benjamini** ^*^
**Axon guidance**	29	1.8	0.0014	0.25
**Calcium signaling pathway**	31	1.9	0.0220	0.89
**Glycan structures - biosynthesis 2**	14	0.9	0.0250	0.81
**Adherens junction**	16	1	0.0260	0.73
**Long-term depression**	16	1	0.0290	0.69
**Wnt signaling pathway**	26	1.6	0.0520	0.84

^*^Benjamini is a measure of family-wise false discovery rate as described by Huang da et al. [13].

### Pathway studio identifies hub genes important to apoptosis and cell cycle

**Figure 1:**
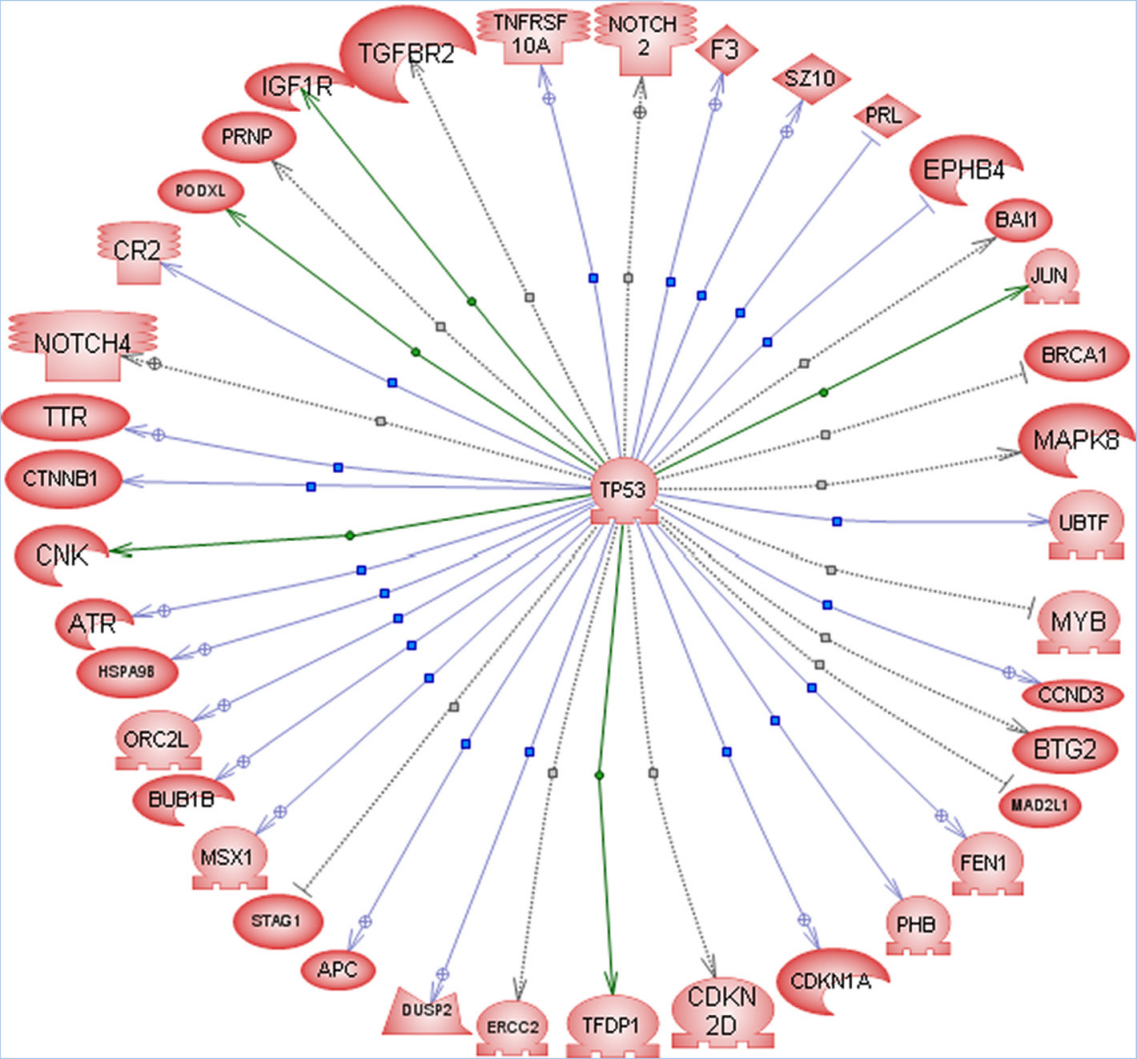
Enriched genes in PRKG2-null growth plate in the P53 pathway. The P53 differentially expressed gene pathway as drawn by pathway studio software. A total of 38 target genes were identified, many of which were also identified as over-enriched for their own downstream targets by Pathway Studio. Additional details regarding the associated targets and publications supporting these target predictions are provided in Additional file 3.

We used Pathway Studio software to search for genes that group into pathways based on published results in PubMed. Many pathways were differentially represented between the two *PRKG2* genotypes. For this reason, we focused our attention on pathways relevant to the known biological function of PRKG2. A sample of highly relevant hub genes indentified by Pathway studio included: cAMP responsive element binding protein 1 (*CREB1*), cyclin-dependent kinase inhibitor 1A (*CDKN1A*/ *P21*/ *Cip1*), catenin (cadherin-associated protein), beta 1, 88 kDa (*CTNNB1*), epidermal growth factor receptor (*EGFR*), E1A binding protein p300 (*EP300*), jun proto-oncogene (*JUN*), tumor protein P53 (*P53*), ras homolog family member A (*RHOA*), and SRC proto-oncogene, non-receptor tyrosine kinase (*SRC*). Each of these genes had many differentially regulated genes (p < 0.05) within their pathway across *PRKG2* genotypes using the common regulators analysis (Additional file 3). The direct regulators analysis also suggested that P21, CTNNB1, EGFR, and JUN pathways were differentially regulated across *PRKG2* genotypes. Additional pathways/hub genes with differentially expressed target genes across genotypes are listed in Additional file 3. A combined analysis of all differentially expressed genes found across PMT levels identified additional target genes in the P53 pathway (n = 476 genes downstream of P53). Figure 1 shows the P53 target genes detected from the PMT 70 gene list by Pathway Studio.

### Real-time PCR results confirmed differential expression of P53 target genes

**Figure 2:**
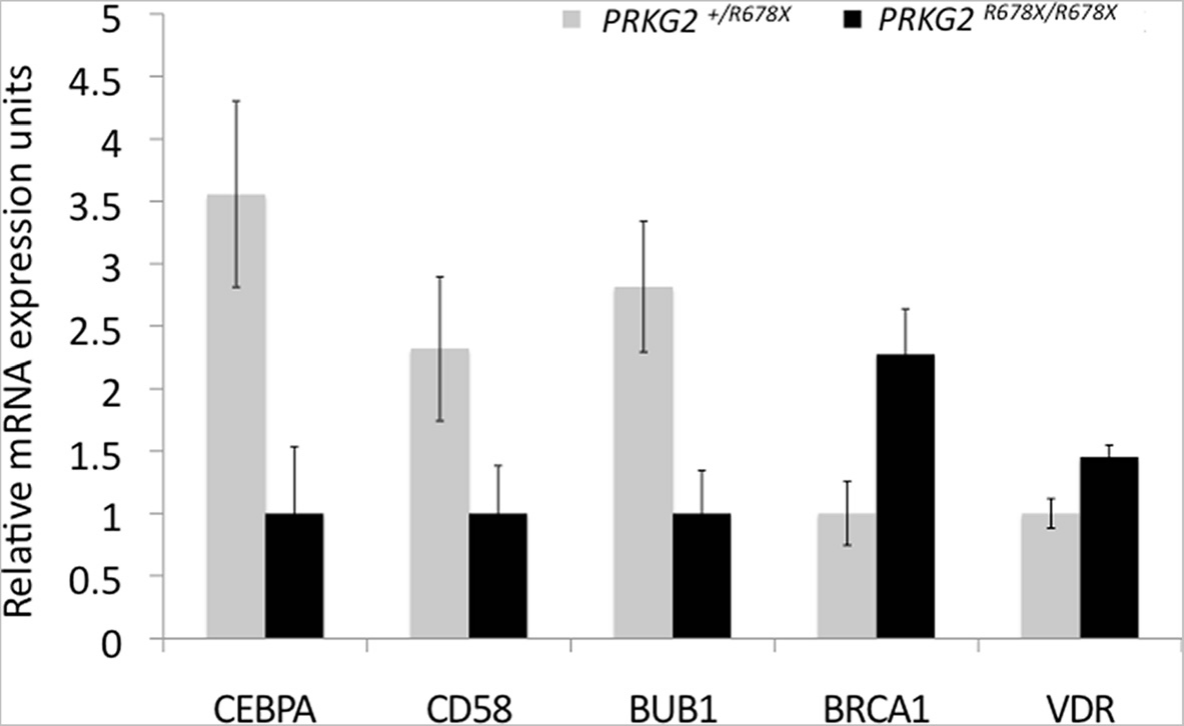
Validation of DE genes in the P53 pathway. The P53 pathway genes validated as differentially expressed by quantitative real-time PCR (p < 0.05). Transcripts *BRCA1*, *BUB1*, *CEBPA*, and *CD58* were found to be differentially expressed using the ddCT method, while *VDR* was differentially expressed when comparing the natural log (ln) starting transcript copy number between genotypes. Fold changes in gene expression are presented, where the genotype with the lowest expression level was set to one to facilitate visualization of fold change differences.

We tested eight genes to validate their status as differentially expressed, including seven genes from the P53 pathway. The primer sequences used in real-time qPCR for these genes are provided in Additional file 4. Five of these seven P53 target genes were confirmed as DE (p < 0.05). Three of these genes (breast cancer 1, early onset (*BRCA1*), CCAAT/ enhancer binding protein (C/EBP), alpha (*CEBPA*), and CD58 molecule (*CD58*)) were detected as DE using the ddCT method while two genes, BUB1 mitotic checkpoint serine/threonine kinase (*BUB1*) and vitamin D (1,25- dihydroxyvitamin D3) receptor (*VDR*), were detected as DE by difference in natural log starting copy number (p < 0.05). Figure 2 presents the coordinated change in expression of these five genes.

## Discussion

### DAVID results indicated that specific transcripts involved in apoptosis, cellular hypertrophy, DNA damage repair, and proliferation are differentially enriched as a result of bovine achondroplasia

The enrichment results from DAVID identified global changes in gene expression related to cell development, proliferation, and death. The most significantly enriched GO biological process for the PMT 70 data was cell development, likely representing genes related to hypertrophy and terminal differentiation. Furthermore, cell proliferation and cell cycle genes were also identified; likely due to the increased proliferative phenotype of PRKG2 null (*PRKG2*^*R678X/R678X*^) chondrocytes. Last, and not unexpected, were changes related to cell death and response to stress. These changes in gene expression are likely related to apoptosis in the growth plate due to uncoordinated development of the growth plate, which results in the inappropriate mixing of hypertrophic and proliferative growth zones. A combined analysis of differentially expressed gene lists across all of the PMT levels resulted in similar, more statistically significant, enrichment of transcripts for biological processes related to PRKG2 function. Enriched biological processes from combined PMT data include: apoptosis, cell hypertrophy/differentiation, cell proliferation and regulation of cell cycle, and processes related to proper organization of the growth plate (cell motility, locomotory behavior and established cell localization). WNT and calcium signaling pathways were over-represented in a KEGG pathway enrichment analysis using the combined gene lists. Additionally, changes were observed in glycan structure processes, likely due to the disorganization of the growth plate in PRKG2 null individuals. A key result from the enrichment analysis across all PMT was the detection of over-represented WNT pathway transcripts, which was recently shown to regulate PRKG2 [5].

The functional gene-clustering tool in DAVID identified modules of genes enriched for actin cytoskeletal and cell motility, apoptosis, kinase/kinase function, proliferation/ cell cycle and metal ion binding from the PMT 70 gene list (ES > 2.9). Combining all of the differentially expressed gene lists across PMT levels resulted in similar results with the addition of DNA damage repair (ES = 1.66) and embryonic appendage/limb development gene clusters (ES = 1.62). The biological functions enriched in these gene clusters are consistent with increased proliferative and altered morphology observed in PRKG2 null (dwarf) vs. wild-type (unaffected) individuals. In summary, DAVID enrichment results are consistent with known biological functions of PRKG2 and phenotypes observed in null animals.

### Pathway studio identified two known PRKG2 targets in CTNNB1 and SRC as well as a host of other possible down-stream targets involved in apoptosis and proliferation

Many over-represented pathways detected by Pathway studio appear to be involved primarily in the control of proliferation, apoptosis or a combination of both processes [14-22]. Gene pathways differentially expressed between genotypes include: *P21*, *CREB1*, *EP300* (*p300*), *EGFR*, *JUN*, *SRC*, *RHOA*, and *CTNNB1*. P21 appears to be largely inhibited or down-regulated in PRKG2 wild-type individuals compared to PRKG2 null individuals. JUN, CREB1, EP300, RHOA, and SRC target genes appear to be up-regulated in PRKG2-null individuals. Beta-catenin (CTNNB1) was previously identified as a downstream target of PRKG2 and an important regulator of bone growth in the WNT pathway [5]. Additionally, it has been suggested that other regulators identified in this study (i.e. EP300 and EGFR) interact with CTNNB1 to regulate cellular differentiation and proliferation respectively [15,21]. SRC is regulated by PRKG2 mediated protein tyrosine phosphatase, non-receptor type 6 (SHP-1) phosphorylation in mechanically stimulated osteoblasts [23]. In turn, SRC acts as a mitogen by promoting cell growth through RAS and the Raf-MEK-ERK pathways. This study also finds a RAS homologue pathway, RHOA, to be differentially represented between the PRKG2 null and unaffected individuals.

### P53 is known to regulate proliferation and apoptosis in the growth plate

The P53 pathway is a logical candidate for PRKG2 regulation because P53 is a key regulator of proliferation, apoptosis and response to DNA damage by regulating target gene expression [24-28]. P53 regulates critical checkpoints in the cell cycle. Increased P53 is known to increase P21 expression, which is essential for progression from G1 to S phase of cell cycle [28]. Notably, P21 is upregulated in a variety of achondroplasia phenotypes in humans [29,30]. The location and time where apoptosis occurs in the growth plate is critical for proper long bone growth. To allow proper proliferation of chondrocytes, P53 is down regulated in the resting zone to prevent apoptosis [31]. At the same time, apoptosis is required for deposition of new bone at the epiphyseal-metaphyseal junction to allow elongation of long bones [32-34]. P53 induced apoptosis may be important to facilitate this process in long bone growth. Consistent with this hypothesis, data indicates that P53 −/− mouse chondrocyte progenitor cells have impaired terminal differentiation and enhanced proliferation as well as progression into osteosarcoma [24,35]. Interestingly, PRKG2 is located underneath a quantitative trait locus on mouse chromosome 5 for alpha-radiation induced osteosarcoma [20]. The connection between PRKG2 and P53 may be GSK3. GSK3 has been shown to regulate P53 via MDM2 proto-oncogene (MDM2) phosphorylation, leading to down regulation of P53 [36].

To validate the changes in P53 target gene’s expression, we confirmed five of seven P53 target genes as differentially expressed by real-time PCR (p < 0.05). Gene expression profiles indicated down-regulation of P53 in PRKG2 null individuals, consistent with increased cellular proliferation. The P53 regulated transcripts *BRCA1*, and *VDR* were confirmed as up-regulated in PRKG2 null individuals, while *BUB1*, *CD58*, *CEBPA* were down-regulated. These results indicate that PRKG2 may act to turn on or turn off P53 either directly or indirectly. Our microarray experiment indicated that MDM2 was significantly (p < 0.001) up regulated in PRKG2 null individuals, but we were unable to find more than a trend towards up-regulation of *MDM2* in PRKG2 nulls (p < 0.20). MDM2 acts as part of a feedback loop to regulate P53, first promoting and then inhibiting P53 expression. No protein or functional data was generated in this experiment to confirm these results.

Since MDM2 phosphorylation down regulates P53 signaling, we asked if MDM2 could be phosphorylated by PRKG2, acting as a direct target of PRKG2 regulation that mediates P53 repression. Using the GPS software [37], we were able to predict several PRKG2 phosphorylation sites on both MDM2 and P53. The maximum log of odds (LOD) score for MDM2 phosphorylation by PRKG2 was 7.0, where LOD = 1.69 is equivalent to p < 0.05. The most significant LOD score for P53 phosphorylation by PRKG2 was 4.67. In comparison, SOX9, a known PRKG2 substrate, had a PRKG2 phosphorylation site predicted with a LOD = 9.33.

## Conclusions

We identified differentially represented pathways in growth plate cartilage due to dwarfism caused by loss of PRKG2 function. Biological processes differentially regulated due to PRKG2 loss of function based on ontology enrichment analysis include: apoptosis, cellular differentiation, organization and proliferation. Hub genes with differentially expressed targets included: *P21*, *CREB1*, *EP300* (*P300*), *EGFR*, *JUN*, *SRC*, *RHOA*, *CTNNB1*, and *P53*. Both SRC and CTNNB1 were previously identified as downstream targets of PRKG2. We validated five of eight DE genes from a microarray experiment by real-time PCR, including five P53 regulated genes, demonstrating that pathway enrichment can identify truly DE genes when no other DE genes are detected. Differential expression of P53 target genes between PRKG2 null and wild-type individuals may indicate another way in which PRKG2 regulates growth plate chondrocyte proliferation and apoptosis. Alternatively, P53 expression across PRKG2 genotypes may vary as a result of cellular response to stress caused by DNA damage and cellular death in cells that are unable to terminally differentiate in a reactive fashion to loss of PRKG2 function. Regardless of the cause of P53 differential expression, the change in P53 target gene expression is important given the role of P53 in growth plate development and regulation of apoptosis. These results demonstrate that important differences in gene expression in response to or as a result of PRKG2 loss of function were identified by pathway enrichment analysis. Identification of differentially enriched pathways due to loss of PRKG2 function may be useful in suggesting potential treatments for achondroplasia and other bone diseases.

## Methods

### Biological samples

Dwarf (*PRKG2*^*R678X*/*R678X*^) and unaffected (*PRKG2*^*R678X*/*+*^) calves were produced by mating a mother, daughter (carrier, dwarf) pair to a single carrier bull. Standard embryo transfer methods were used to allow implantation into surrogate mothers. More than twenty embryos were produced; however, only six survived to 60 days post-implantation. Six calves, three from each female, were born full term from surrogate mothers in two calving groups. Calves within the two groups were born during the same week and were reared and euthanized together. Two dwarves and one unaffected individual were born in each calving group. Each animal was genotyped to verify the dwarf or unaffected phenotype [3]. All calf rearing, experimental procedures, tissue collection and euthanization were carried out under the guidelines of the Iowa State University Institutional Animal Care and Use Committee under approved protocol number 6-04-5697-B-1.

### Tissue collection

Calves were euthanized at approximately 210 days of age during a period of rapid long bone growth [38]. Whole growth plate was collected from the tibia bone of all the animals and preserved using RNAlater™ (Ambion, Austin, TX). Each tibia was sliced horizontally at the growth plate to allow cartilage pieces to be removed using a sterile razor blade. Extra care was taken to avoid harvest of bone marrow and osteoblasts by leaving a thin layer of growth plate on the bone epiphysis and diaphysis. Samples were archived at - 80°C until processed for RNA isolation.

### RNA isolation

RNA was isolated from growth plate cartilage using TRIzol™ (Invitrogen, Carlsbad, CA). A mass of 0.15 to 0.20 g of growth plate cartilage was placed into a sterile Petri dish with one milliliter (ml) of TRIzol™. Next, each sample was diced into a slurry using a sterile scalpel. Then, samples were homogenized using a rotor star probe in a total volume of 6 ml TRIzol™. Sample yield was measured by spectrophotometer. These initial RNA yield values were used to estimate how much purified RNA could be eluted by column purification (Qiagen, Valencia, CA). Column purified RNA was stored at - 80°C for future use.

### Microarray analysis

An Oligonucleotide 70 mer microarray created by the bovine microarray consortium was used to assay the expression of approximately 24,000 transcripts [39]. Slides were UV cross-linked prior to each hybridization. Synthesis of cDNA was carried out using Superscript II reverse transcriptase (Invitrogen, Carlsbad, CA) along with the Genisphere 3DNA 350 HS kit components (Genisphere, Hatfield, PA). Unaffected animals, *PRKG2*^*R678X/+*^, were compared within calving group to each of the two dwarf, *PRKG2*^*R678X/R678X*^*,* calves, resulting in two microarrays per calving group. Cy 3 and Cy 5 dyes were used for differential display of cDNA binding between genotype treatments to the array. Dye swapping was used for genotype comparisons to account for differential dye incorporation. A total of four microarrays were hybridized to compare *PRKG2*^*R678X/R678X*^ and *PRKG2*^*R678X/+*^ animals using the Genisphere kit. Microarrays were hybridized using GE Healthcare's Lucidea SlidePro hybridization oven (General Electric Healthcare, Piscataway, NJ) at the Iowa State University DNA facility according to manufacturer’s protocol. All microarray data was submitted to NCBI GEO under accession number GSE31627.

### Scanning

Hybridized slides were scanned for visualization of Cy 3 and Cy 5 signal using a Pro Scan Array HT scanner at the microarray facility within the Iowa State University, Plant Sciences Institute. Slides were scanned at three different laser PMT intensities, 70, 80 and 90 PMT, to account for high and low expression levels of various transcripts [40] as different genes will appear as expressed or not expressed at the different PMT levels. Images were analyzed to obtain raw signal intensities and localized background signals with Imagene 5.1 software. Text files were produced for each slide channel for statistical analysis.

### Statistical analysis

Image files were normalized by channel to correct for background, differential dye incorporation using Lowess normalization [41], and heterogeneous variance by median centering and natural log transformation using R scripts kindly provided by Dr. Dan Nettleton, Iowa State University. Data were analyzed using PROC MIXED of SAS accounting for fixed effects of the dye, genotype and calving group. The slide was included as a random effect. To account for random effects having an estimated variance of zero, we used the Kenworth-Rodgers correction. Multiple testing corrections were made by calculating q-values to determine false discovery rate (FDR) levels for each gene list [42].

### Pathway and gene function analysis

Genes on the bovine microarray consortium 70-mer array were annotated where annotation or BLAST analysis could not identify the gene corresponding to each probe. We used the SWISS-PROT protein IDs to perform analysis of gene function and search for over-represented gene pathways using DAVID [13,43] and Pathway Studio (Ariande Genomics, Rockville, MD). Gene expression analysis with DAVID identified over-represented gene categories, genes of specific molecular function, functional gene clusters and pathways. Pathways identified by DAVID were mined from the KEGG [44] and Biocarta pathway analysis tools. The default settings for EASE were used for all of these analyses. Pathway studio was used to find specific gene pathways that were highly represented within a gene list. Pathway studio uses a wave propagation algorithm to text mine databases (i.e. curated literature data from PubMed) for information about groups of entities used in analysis and tries to relate these groups back to the gene list. We used the direct regulators (relationship between genes on a gene list) and common regulators (identifies upstream regulators that have targets within a gene list) analyses. Gene pathways were selected as differentially represented based on the number of connections originating from a common regulator that differed between the two genotypes.

### Real-time PCR

The cDNA from growth plate cartilage was analyzed for differential expression by replicating each sample three times for the target and housekeeping gene in two separate plate replicates (i.e. six total replicates of both the target and housekeeping gene) using primers listed in Additional file 4 for the following seven P53 regulated genes*: BRCA1, BUB1 CEBPA, CD58, MDM2, PRL and VDR*. In addition, the SOX9 responsive gene, phosphate regulating endopeptidase homolog, X-linked (PHEX), was assayed across genotypes by real-time PCR. We used beta-actin as our housekeeping gene. Primer template sequences were retrieved using the 3’ human exonic sequence as a BLAST template to find the bovine template sequence. Genes analyzed by real-time PCR were selected based on the Pathway Studio analysis. Eight target genes and one house keeping gene (Beta-actin) were analyzed to determine the validity of microarray gene expression for the P53 pathway. Primers, PMT levels, and thermocycling protocols are listed in Additional file 4. Standard curves for each target and reference gene were made and evaluated for linearity in amplification. All real-time PCR data were analyzed using MyiQ™ software to check for primer dimmer, linear amplification (BioRad; Hercules, CA).

### Statistical analysis of real-time PCR

Real-time PCR data was analyzed using the delta-delta cycle threshold (ddCt) method [45] in Proc GLM of SAS (SAS Institute; Cary, NC). The model used to analyze the expression data was

$$ {\mathrm{y}}_{\mathrm{i}\mathrm{j}\mathrm{kl}} = {\mathrm{p}}_{\mathrm{i}} + {\mathrm{g}}_{\mathrm{i}\mathrm{j}} + {\mathrm{g}\mathrm{r}}_{\mathrm{k}} + {\mathrm{e}}_{\mathrm{i}\mathrm{j}\mathrm{kl}}, $$

where y = ddCt (i.e. the target gene – housekeeping gene (beta-actin) for the dwarf subtracted from the target gene– housekeeping gene for an unaffected individual), p = real-time plate replicate, g = genotype, gr = common calving and rearing group, and e = error. When genes were not detected as differentially expressed using the ddCT method, we analyzed the difference in log starting copy number where the copy number of each target gene was compared against a standard curve for the same transcript. The model used to analyze the natural log (ln) starting copy number data was

$$ {\mathrm{y}}_{\mathrm{i}\mathrm{j}\mathrm{kl}}={\mathrm{p}}_{\mathrm{i}}+{\mathrm{g}}_{\mathrm{i}\mathrm{j}}+\mathrm{g}{\mathrm{r}}_{\mathrm{k}}+{\mathrm{e}}_{\mathrm{i}\mathrm{j}\mathrm{kl}}, $$

where y = ln starting value of the target gene mRNA adjusted for ln starting value of housekeeping gene mRNA (ln starting number target – ln starting number *beta-actin*), p = real-time plate replicate, g = *R678X PRKG2* genotype, gr = common calving and rearing group, and e = error.

## Additional files

Additional file 1:
**List of annotations for all putative DE genes and enriched pathways.** Additional file 1 contains all probe and annotation information for the cDNA microarray used in this study. Specifically, the data is split into worksheets by the specific analysis and annotation information. Each work sheet contains probe IDs differentially expressed at p<0.05 at one of three different PMT measures. SwissProt IDs are the human or mouse IDs for the corresponding bovine transcript. Gene names from DAVID are the corresponding gene names from Swissprot as Swissprot IDs were used to run the DAVID analysis. Additional file 2:
**Gene Ontology (GO) enrichment results.** Additional file 2 contains all GO enrichment results for the DE genes in the PMT70 and combined datasets. Additional file 3:
**Pathway studio enrichment results divided by up and down regulated genes in PRKG2 null (dwarf) animals.** Additional file 3 contains all Pathway studio pathway enrichment results. It is divided into worksheets by the two analysis methods detecting direct or common regulators and the entities which provide descriptions of the curated regulators. In addition, the data is divided by which genes were up regulated in the wild**-**type (*PRKG2* ^*+/-*^) or the dwarf (*PRKG2* ^*-/-*^) genotypes. Additional file 4:
**Primer sequences used to confirm differential expression of P53 target genes by real-time qPCR.** Additional file 4 provides a table of P53 target genes and their corresponding primer sequences used in real-time qPCR analysis.

## Abbreviations

BLAST: Basic local alignment search tool
BRCA1: Breast cancer 1, early onset
BUB1: BUB1 Mitotic checkpoint serine/threonine kinase
CD58: CD58 Molecule
CDKN1A: Cyclin-dependent kinase inhibitor 1A (p21, Cip1)
cDNA: Complimentary deoxyribonucleic acid
CEBPA: CCAAT/ Enhancer binding protein (C/EBP), alpha
cGMP: Cyclic guanosine monophosphate
CNP: C-type naturetic peptide
COL2: Collagen 2
COL10: Collagen 10
CREB1: cAMP Responsive element binding protein 1
CTNNB1: Catenin (cadherin-associated protein), beta 1, 88 kDa
DAVID: Database for annotation, visualization and integrated discovery
DE: Differentially expressed
DNA: Deoxyribonucleic acid
EGFR: Epidermal growth factor recptor
EP300: E1A Binding protein p300
ES: Enrichment score
FGFR3: Fibroblast growth factor receptor 3
FWFDR: Family-wise false discovery rate
GO: Gene ontology
GSK3B: Glycogen synthase 3 beta
JNK: Mitogen-activated protein kinase 8 (MAPK8)
JUN: Jun proto-oncogene
KEGG: Kyoto encyclopedia of genes and genomes
MAPK: Mitogen-activated protein kinase
MDM2: MDM2 proto-oncogene, E3 ubiquitin protein ligase
MHC: Major histocompatability complex
Muc1: Mucin 1, cell surface associated
P53: Tumor protein P53
NF-κB: Nuclear factor of kappa light polypeptide gene enhancer in B-cells
NO: Nitric oxide
PCR: Polymerase chain reaction
PHEX: Phosphate regulating endopeptidase homolog, X-linked
PMT: Photomechanical transfer unit
PRKG2: cGMP-dependent, type II, protein kinase
PRL: Prolactin
PYK2: Protein tyrosine kinase 2 beta
RHOA: Ras homolog family member A
RNA: Ribonucleic acid
SHP-1: Protein tyrosine phosphatase, non-receptor type 6 (PTPN6)
SOX9: SRY, sex determining region Y, −box 9
SRC: SRC proto-oncogene, non-receptor tyrosine kinase
VDR: Vitamin D (1,25- dihydroxyvitamin D3) receptor
VEGF: Vascular endothelial growth factor
WNT: Wingless-type MMTV integration site family
