# Supplementary material for: Transcriptional profiling of PRKG2-null growth plate identifies putative down-stream targets of PRKG2
Source: BMC Res Notes. 2015 Apr 30;8:177. doi: 10.1186/s13104-015-1136-6 (PMC4419418; doi:10.1186/s13104-015-1136-6)
Supplement: Additional file 4: — Primer sequences used to confirm differential expression of P53 target genes by real-time qPCR. Additional file 4 provides a table of P53 target genes and their corresponding primer sequences used in real-time qPCR analysis. [file 13104_2015_1136_MOESM4_ESM.docx]

## Primer sequences used to confirm differential expression of P53 target genes by real-time qPCR.

| **Gene names** | **PMT^1^** | **Pathway^2^** | **Primer Sequence (5’->3’)^3, 4^** |
| --- | --- | --- | --- |
| *B-actin* | N/A | Housekeeping | **F-** AAGGACTCGTACGTGGGGGATGA |
|  |  | gene | **R-** AAGGACTCGTACGTGGGGGATGA |
| *BRCA1* | 70 | *TP53* | **F-** CCAGAAAAGGCAAAGACTGC |
|  |  |  | **R-** TGCTTGTCTCCTGAATGACG |
| *BUB1* | 70 | *TP53* | **F-** TTATTCAGCCCACCTGTTCC |
|  |  |  | **R-** TCTCCGTGGATGATTTCACA |
| *CD58* | 80 | *TP53* | **F-** TGAGTGCCGAAGAACTGATG |
|  |  |  | **R-** TATCGTCCCCTTCTCCTCCT |
| *CEBPA* | 70* | *TP53* | **F-** CTGGCTTTATCGGGATCTCA |
|  |  |  | **R-** TAACCCTGTGCCTTGGAAAC |
| *MDM2* | 90 | *TP53* | **F-** GAAATGAATCCTCCCCTTCC |
|  |  |  | **R-** GACATCAAAGCCCTCGTCTT |
| *PHEX* | 70 | *SOX9* | **F-** GTCGGTGCTTAGGTTGGAAA |
|  |  |  | **R-** GTATTGAGGGACTCGGACCA |
| *PRL* | 70 | *BRCA1* | **F-** ACCCTGTGTGGTCAGGACTC |
|  |  |  | **R-** TGTGGGCTTAGCAGTTGTTG |
| *VDR* | 90 | *TP53* | **F-** ACTCCGATGACCCTTCTGTG |
|  |  |  | **R-** TCAATGGCACTCGACTTCAG |

^1^PMT refers to the PMT level gene expression measurement each gene was deemed significant (p<0.05).

^2^Pathway refers to the gene regulation list each gene was related with using Pathway Studio

^3^F and R are forward (sense) and reverse (antisense) oligos

^4^All primers were amplified using the same protocol: 1) 95C for 10 min, 2) 95C for 15 s, 3) 60C for 30 s, 4) Repeat steps 2 and 3, 40 times, 5) 60C + 0.5C/cycle for 70 cycles, 6) end.

**CEBPA* was found as a common regulator on the PMT70 gene list.
